# GLUcose COntrol Safety & Efficacy in type 2 DIabetes, a systematic review and NETwork meta-analysis

**DOI:** 10.1371/journal.pone.0217701

**Published:** 2019-06-25

**Authors:** Guillaume Grenet, Shams Ribault, Giao Bao Nguyen, Faustine Glais, Augustin Metge, Thomas Linet, Behrouz Kassai-Koupai, Catherine Cornu, Théodora Bejan-Angoulvant, Sylvie Erpeldinger, Rémy Boussageon, Aurore Gouraud, Fabrice Bonnet, Michel Cucherat, Philippe Moulin, François Gueyffier

**Affiliations:** 1 Service de Pharmacotoxicologie, Hospices Civils de Lyon, Lyon, France; 2 Université Lyon 1, CNRS, UMR5558, Laboratoire de Biométrie et Biologie Evolutive, Lyon, France; 3 Université de Lyon, Lyon, France; 4 CIC1407 INSERM, Lyon, France; 5 CHRU de Tours, Service de Pharmacologie Médicale—Tours, France; 6 Université de Tours, Groupe Innovation & Ciblage Cellulaire, équipe Pharmacologie des Anticorps Thérapeutiques chez l’Homme–Tours, France; 7 Département de médecine générale, Université Lyon 1—Lyon, France; 8 Département de Médecine Générale, Faculté de Médecine et de Pharmacie, Université de Poitiers—Poitiers, France; 9 CHU Rennes, Université de Rennes 1—Rennes, France; 10 Fédération d’endocrinologie, maladies métaboliques, diabète et nutrition, INSERM UMR 1060 CARMEN Hospices Civils de Lyon, Université Lyon 1- Lyon, France; University of Oxford, UNITED KINGDOM

## Abstract

**Background:**

The last international consensus on the management of type 2 diabetes (T2D) recommends SGLT-2 inhibitors or GLP-1 agonists for patients with clinical cardiovascular (CV) disease; metformin remains the first-line glucose lowering medication. Last studies suggested beneficial effects of SGLT-2 inhibitors or GLP-1 agonists compared to DPP-4 inhibitors, in secondary CV prevention. Recently, a potential benefit of SGLT-2 inhibitors in primary CV prevention also has been suggested. However, no comparison of all the new and the old hypoglycemic drugs is available on CV outcomes. We aimed to compare the effects of old and new hypoglycemic drugs in T2D, on major adverse cardiovascular events (MACE) and mortality.

**Methods and findings:**

We conducted a systematic review and network meta-analysis of clinical trials. Randomized trials, blinded or not, assessing contemporary hypoglycemic drugs on mortality or MACE in patients with T2D, were searched for in Medline, the Cochrane Central Register of Controlled Trials (CENTRAL), and ClinicalTrials.gov. References screening and data extraction were done by multiple observers. Each drug was analyzed according to its therapeutic class. A random Bayesian network meta-analysis model was used. The primary outcomes were overall mortality, cardiovascular mortality, and MACE. Severe adverse events and severe hypoglycemia were also recorded.

175,966 patients in 34 trials from 1970 to 2018 were included. No trials evaluating glinides or alpha glucosidase inhibitors were found. 17 trials included a majority of patients with previous cardiovascular history, 16 trials a majority of patients without. Compared to control, SGLT-2 inhibitors were associated with a decreased risk of overall mortality (OR = 0.84 [95% CrI: 0.74; 0.95]), SGLT-2 inhibitors and GLP-1 agonists with a decreased risk of MACE (OR = 0.89 [95% CrI: 0.81; 0.98] and OR = 0.88 [95% CrI: 0.81; 0.95], respectively). Compared to DPP-4 inhibitors, SGLT-2 inhibitors were associated with a decreased risk of overall mortality (OR = 0.82 [95% CrI: 0.69; 0.98]), GLP-1 agonists with a decreased risk of MACE (OR = 0.88 [95% CrI: 0.79; 0.99]). Insulin was also associated with an increased risk of MACE compared to GLP-1 agonists (OR = 1.19 [95% CrI: 1.01; 1.42]). Insulin and sulfonylureas were associated with an increased risk of severe hypoglycemia. In the trials including a majority of patients without previous CV history, the comparisons of SGLT-2 inhibitors, metformin and control did not showed significant differences on primary outcomes. We limited our analysis at the therapeutic class level.

**Conclusions:**

SGLT-2 inhibitors and GLP-1 agonists have the most beneficial effects, especially in T2D patients with previous CV diseases. Direct comparisons of SGLT-2 inhibitors, GLP-1 agonists and metformin are needed, notably in primary CV prevention.

**Trial registration:**

PROSPERO CRD42016043823.

## Introduction

Type 2 diabetes (T2D) is a public health issue, with a dramatically increasing incidence in the world. Cardiovascular diseases (CVD) are the main cause of mortality in T2D patients. Many hypoglycemic drugs are currently available; their benefits have been evaluated with conflicting results. Network meta-analysis allows several treatments to be compared through direct and indirect comparisons. Previous network meta analyses on hypoglycemic drugs were focused on intermediate outcomes, such as glycated hemoglobin (HbA1c), or did not compare the effect of the drugs on mortality or major adverse cardiovascular events (MACE) in the absence of data [1]. Since then, new clinical trials assessing SGLT-2 inhibitors or GLP-1 receptor agonists showed promising results on mortality or on cardiovascular outcomes (EMPAREG-OUTCOME [2], CANVAS-Program [3], LEADER [4], SUSTAIN-6 [5]), allowing Zheng et al to show a lower mortality rate with SGLT-2 inhibitors or GLP-1 receptor agonists compared to control or DPP-4 inhibitors, mainly in secondary cardiovascular prevention [6]. The last international consensus recommends SGLT-2 inhibitors or GLP-1 receptor agonists for patients with clinical cardiovascular disease; metformin remains the first-line therapy for glucose lowering medication [7]. However, the last cardiovascular outcome trial assessing a GLP-1 receptor agonists did not showed a decreased risk of overall mortality [8]. Following the recently published DECLARE TIMI 58 trial [9], a meta-analysis suggested a potential benefit of SGLT-2 inhibitors in primary cardiovascular prevention, but did not include GLP-1 receptor agonists or metformin [10]. Most of hypoglycemic drugs have not been directly compared in head to head clinical trials. Up to now, no comparison of all the new and the old hypoglycemic drugs is available on major cardiovascular outcomes. The purpose of this study was to compare all the currently available hypoglycemic drug classes on major adverse cardiovascular events (MACE) and on mortality in patients with T2D, through a network meta-analysis approach of randomized clinical trials.

### Protocol registration number

PROSPERO CRD42016043823

## Methods

Methods have been previously described [11]. This meta-analysis was conducted following the Preferred Reporting Items for Systematic Reviews and Meta-Analyses (PRISMA) statement and its extension for reviews incorporating network meta-analyses (S1 Fig) [12].

### Search strategy and selection criteria

Randomized clinical trials (RCTs), double-blind or open, including patients with type 2 diabetes, evaluating a specific contemporary hypoglycemic drug through clinically relevant outcomes (as primary or secondary outcomes) have been included. Clinically relevant outcomes considered here were: overall mortality, cardiovascular mortality, MACE (myocardial infarction–MI–, acute coronary syndrome, or stroke) and diabetic microangiopathy (new or worsening) that is clinically symptomatic or leading to a therapeutic intervention such as surgery, photocoagulation, or dialysis. Trials which used drugs which have been withdrawn from the market (such as phenphormin and tolbutamide) were not included. Trials comparing drugs of the same therapeutic class and glucose lowering treatment intensifications without specific drugs were excluded.

English language published trials were searched in PubMed and Central databases, without time restriction, up to March 2016 (see S1 Table). Unpublished and other on-going trials were searched through references of published meta-analyses, ClinicalTrials.gov, congress abstracts. On-going trials of potential interest were followed until November 2018 for final results. The study selection, data extraction and risk of bias assessment were performed by at least two independent reviewers (GG and SR, GN, FaG, AG or TL), consensus was reached in the case of disagreements. Studies were first screened on the basis of their titles and abstracts, then included based on the full text. The quality of the studies was assessed using the Cochrane Collaboration’s tool for assessing risk of bias in RCTs [13]. Summary estimates of the treatment effect and summary of patients’ characteristics (age, gender, cardiovascular risk factors) were extracted.

### Outcomes of the meta-analysis

Primary outcomes of this analysis were: overall mortality, cardiovascular mortality, and major adverse cardiovascular events (MACE: cardiovascular death, non-fatal MI, and non-fatal stroke), as described in the protocol [11]. For MACE, proxies have been used for 10 studies among the 27 trials with available data (see S2 Table). Diabetic microangiopathy was a pre-specified secondary outcome, but its reporting in the included studies was heterogeneous and not available in many studies. Instead, detailed results on macrovascular outcomes (all and non-fatal MI, all and non-fatal stroke) were retrieved. Serious adverse events and severe hypoglycemia were also reported as secondary outcomes. For Serious adverse events, reported definitions are presented in supplementary S3 Table.

### Data analysis

Each drug (including each drug dose) was analyzed according to its therapeutic class: biguanide (metformin), alpha glucosidase inhibitors, sulfonylureas, glitazones, glinides, insulin, DPP-4 inhibitors, GLP-1 receptor agonists, and SGLT-2 inhibitors. Placebo, diet control and active control without specific drug classes were considered together as control treatment. A random Bayesian network meta-analysis model was used [14]. The prior distribution was chosen as non-informative, the posterior distribution was estimated using Markov Chain Monte Carlo method [15]. The treatment effect estimate was presented for the network estimation and for the direct comparison, when available, through odds ratio (OR) and its 95% credible interval (95% CrI). Ranking probability and the surface under the cumulative ranking (SUCRA) values were estimated for ranking the drug classes [16]. Sensitivity analyses after considering only double-blind studies and according to two potential effect modifiers, high versus low baseline cardiovascular risk and high versus low glycemic contrast during the study, were conducted. The level of baseline cardiovascular risk of the trial was defined using the proportion of subjects with previous cardiovascular events. Trials below the mean proportion across all trials defined the subset of trials of ‘low cardiovascular risk’; trials above the mean proportion defined the subset of trials of ‘high cardiovascular risk’. Glycemic contrast during the study was defined by the HbA1c difference across arms of the trial. Trials below the mean HbA1c difference across all trials defined the subset of trials of “low glycemic contrast”, trials above the mean HbA1c difference defined the subset of trials of “high glycemic contrast”. Heterogeneity was analyzed using the I^2^. Inconsistency of the network was searched for, using the Node-splitting analysis of inconsistency of the Gemtc package [17]. Analyses have been conducted using R [18] (version 3.3.1) and JAGS [19] with the Gemtc package [17] (version 0.8–2). Meta package [20] was used to illustrate the treatment effect at the trial level.

## Results

### Bibliographic search and included trials

**Fig 1:**
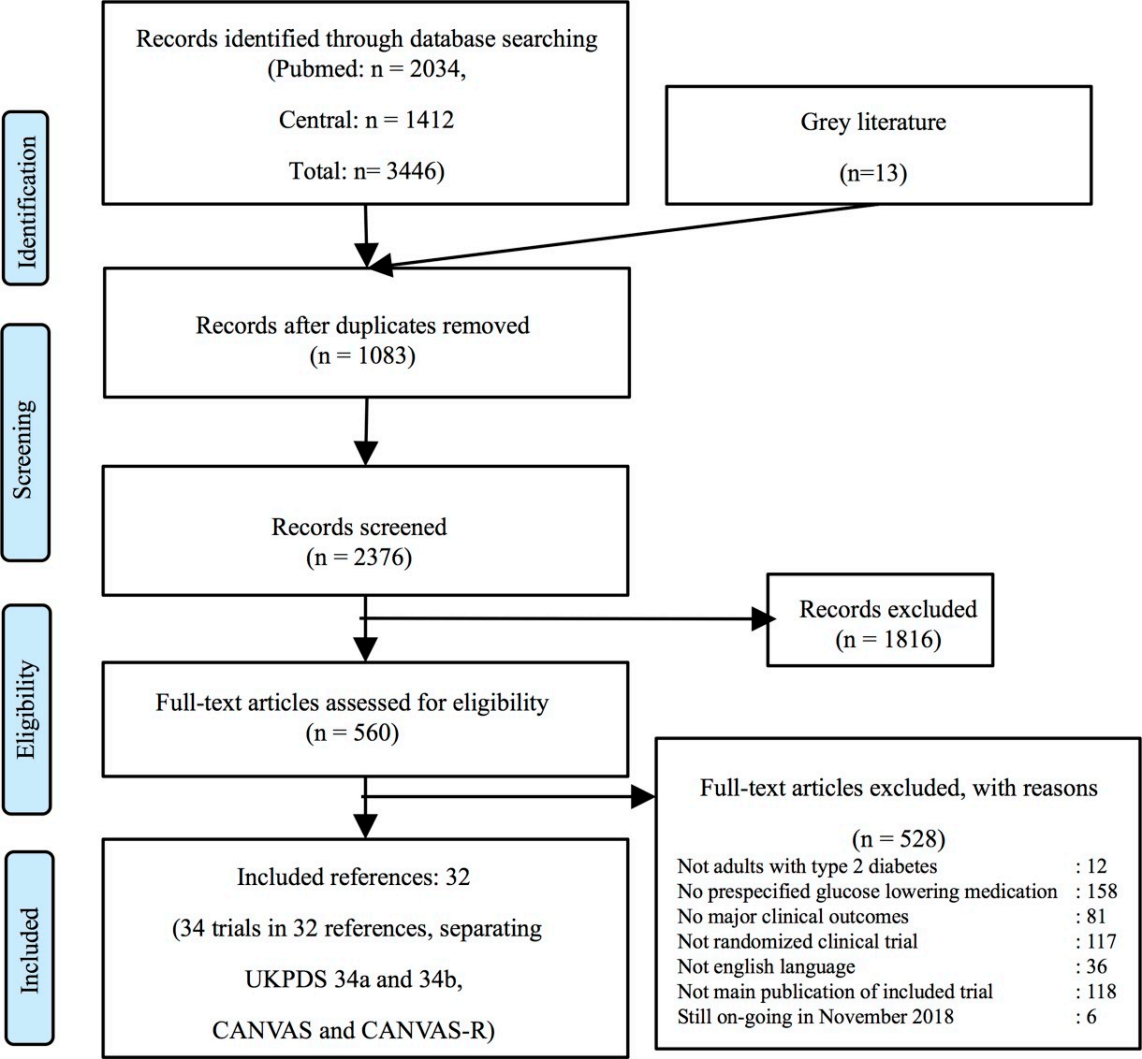
Flow diagram of bibliographic search (following PRISMA guidelines).

The bibliographic search retrieved 3,459 citations. The selection process is presented in Fig 1. Thirty-four trials with 175,966 patients were included [2–5, 8, 9, 21–46]. We did not retrieve trials evaluating alpha glucose inhibitors or glinides. UKPDS34 [25] was considered as two trials, UKPDS34a and UKPDS34b [47]. For UGDP [23], UKPDS33 [24] and TIDE [37] trials, arms with the same drug class were summed up. For UGDP, the tolbutamide group was not included. For the ORIGIN study [36], in which more than 80% of subjects had T2D, only data from the T2D sub-group were used when available, data of the whole trial otherwise. The CANVAS-program [3] was considered as two trials, CANVAS and CANVAS-R. Indeed, given a marked difference in the baseline risk between the two cohorts, their pooling was subject to the Simpson’s paradox. We were unable to obtain results of the PPAR study [21] despite having contacted the authors. Data of the recent trial CARMELINA were limited to the public information [22].

Baseline characteristics of included trials are presented in Table 1. Included trials were published over a span of 48 years (from 1970 to 2018). Percentage of males ranged from 29 to 77.6%, percentage of patients i) with high blood pressure or receiving antihypertensive drugs ranged from 11.6 to 95.1%, ii) with dyslipidemia or receiving statins treatment ranged from 0.1 to 92.8% (low use of lipid lowering drugs in UKPDS), iii) receiving antiplatelet treatment ranged from 40.2 to 98.3%, and percentage of current smokers at inclusion ranged from 10.2 to 49.6%. Mean age ranged from 53+/-8.5 to 69+/-7.1 years, mean duration of diabetes from around 0 (UKPDS) to 14.7+/-9.5 years, mean HbA1c at inclusion from 6.3+/-1.3 to 8.8+/-1.7%, mean body mass index (BMI) at inclusion from 23.9+/-3.1 to 32.5+/-6.3 kg.m^-2^. 20 (59%) trials were double-blinded. The summary of the risk of bias assessments and details for each study are presented in supplementary S2 Fig. Only 12 and seven trials provided details on clinical retinopathy and clinical nephropathy, respectively (18 trials for nephropathy when including biological outcomes).

**Table 1:** Baseline characteristics of included trials.

	Year of publication	Blinding	Male	HBP	Lipd	APT	Smoker	Age (year)	Diabetes duration (year)	HbA1c	BMI
**DPP-4_I VERSUS CONTROL**											
**CARMELINA** [22]	2018	DB	62.9	95.1	71.8	68.3	10.2	65.8 (9.1)	14.7 (9.5)	7.9 (1.0)	31.3 (5.3)
**EXAMINE** [35]	2013	DB	67.8	83	90.4	97.2	13.7	61	7.2	8 (1.1)	28.7
**SAVOR.TIMI.53** [40]	2013	DB	66.9	81.8	71.2	75.2	13.4	65 (8.6)	10.3	8 (1.4)	31.1 (5.6)
**TECOS** [42]	2015	DB	70.7	78.4	79.5	78.2	11.4	65.5 (8)	11.6 (8.1)	7.2 (0.5)	30.2 (5.6)
**GLITAZONES VERSUS CONTROL**											
**J.SPIRIT** [44]	2015	O	NA	NA	NA	NA	NA	NA	NA	NA	NA
**Kaku.2009** [32]	2009	O	62.5	69	71	NA	45.2	58	NA	7.6	26.7
**Lee.2013** [39]	2013	NA	73.6	57	73.5	98.3	49.6	61.1 (9.1)	5.8 (6.7)	7.8 (1.7)	23.9 (3.1)
**PROactive** [27]	2005	DB	66.1	75.4	42.9	83.9	13.8	61.8	8	8.1 (1.41)	30.9 (4.8)
**PROFIT.J** [41]	2014	O	64.6	60.8	43.6	NA	NA	69 (7.1)	11.3 (8.9)	7.4 (0.9)	24.2 (3.3)
**RECORD** [31]	2009	O	51.6	65.6	34.1	NA	15.7	58.4 (8.2)	7.1 (4.9)	7.9 (0.7)	31.5 (4.7)
**TIDE** [37]	2012	DB	58.8	88.2	76.4	55.4	12.5	66.4 (6.6)	8.8 (6.8)	7.4 (0.9)	30.6 (5.3)
**GLP-1_A VERSUS CONTROL**											
**ELIXA** [43]	2015	DB	69.3	76.3	92.8	97.5	11.7	60.2 (9.6)	9.3 (8.2)	7.7 (1.3)	30.1 (5.7)
**EXSCEL** [45]	2017	DB	62	90.3	73.5	63.6	11.6	62*	12 (7;18)*	8(7.3;8.9)*	31.8*
**HARMONY** [8]	2018	DB	69	86.5	84.1	77.1	15.8	64.2 (8.7)	14.1 (8.8)	8.7 (1.5)	32.3 (5.9)
**LEADER** [4]	2016	DB	64.2	92.3	75.6	67.7	NA	64.3 (7.2)	12.8	8.7	32.5 (6.3)
**SUSTAIN.6** [5]	2016	DB	60.7	93.5	76.5	NA	NA	64.6 (7.4)	13.9 (8.1)	8.7 (1.5)	NA
**INSULIN VERSUS CONTROL**											
**ORIGIN** [36]	2012	O	65	79.5	53.8	69.2	12.4	63.5 (7.8)	5.4 (6)	6.4	29.9 (5.2)
**UGDP** [23]	1970	NA	29	32.3	13.1	NA	NA	NA	NA	NA	NA
**UKPDS.33** [24]	1998	O	62	11.6	0.1	NA	31	54 (8)	0	6.3 (1.3)	27.3 (5.1)
**METFORMIN VERSUS CONTROL**											
**COSMIC** [26]	2005	O	49.4	NA	NA	NA	NA	58.5 (13)	4.8 (6)	NA	NA
**HOME** [33]	2009	DB	45.6	43	16.1	NA	24.9	61.5 (10.5)	NA	7.9 (1.2)	30 (5)
**UKPDS.34a** [25]	1998	O	46.5	15.5	0.2	NA	25	53 (8.5)	0	7.2 (1.5)	31.7 (4.8)
**UKPDS.34b** [25]	1998	O	60	24.5	0.2	NA	26.5	58.5 (8.5)	0	7.5 (1.8)	29.5 (5.5)
**SGLT-2_I VERSUS CONTROL**											
**CANVAS** [3]	2017	DB	66.1	87.6	72.3	71.6	17.9	62.4 (8)	13.4 (7.5)	8.2 (0.9)	32.1 (6.2)
**CANVASR** [3]	2017	DB	62.8	91.7	76.9	75.1	17.7	64 (8.4)	13.7 (7.9)	8.3 (1)	31.9 (5.7)
**DECLARE.TIMI.58** [9]	2018	DB	62.6	NA	75	61.1	NA	64 (6.8)	11 (6;16)*	8.3 (1.2)	32 (6)
**EMPAREG** [2]	2015	DB	71.4	95	81.1	NA	NA	63.1 (8.7)	NA	8.1 (0.8)	30.6 (5.2)
**SULFONYLUREA VERSUS CONTROL**											
**ADVANCE** [30]	2008	O	57.5	75.1	NA	NA	NA	66 (6)	8 (6.3)	7.5 (1.6)	28 (5)
**UKPDS.33** [24]	(see previous description)										
**GLITAZONES VERSUS SULFONYLUREA**											
**APPROACH** [34]	2010	DB	67.9	80.1	75.9	83.2	16.6	61 (8.7)	4.8	7.2 (0.8)	29.6 (5.4)
**PERISCOPE** [29]	2008	DB	67.4	86.8	81.2	90.1	15.3	59.9 (9.2)	5.9	7.4 (1)	32 (5.2)
**Giles.2008** [28]	2008	DB	73.6	NA	NA	NA	NA	63.8 (9.7)	11.8 (9.3)	8.8 (1.7)	29.6 (5.3)
**PPAR.Study** [21]	NA	O	NA	NA	NA	NA	NA	NA	NA	NA	NA
**TOSCA.IT** [46]	2017	O	58.5	70	57.3	40.2	17.6	62.3 (6.5)	8.4 (5.7)	7.7 (0.5)	30.3 (4.5)
**INSULIN VERSUS SULFONYLUREA**											
**UKPDS.33** [24]	(see previous description)										
**METFORMIN VERSUS SULFONYLUREA**											
**SPREAD.DIMCAD** [38]	2013	DB	77.6	69.4	63.9	83.5	37.5	63.3	5.6 (5.1)	7.6 (1.7)	25.1 (3)

“_i” stands for inhibitor, “_a” stands for agonist. Percentages from the whole trial (or the mean of the arms if not available) for high blood pressure or antihypertensive drugs (HBP), dyslipidemia or statines (Lipd), antiplatelet treatment (APT) and current smoker (Smoker); mean and standard deviation from the whole trial (or the mean of the arms if not available) for age, diabetes duration, baseline HbA1c and baseline body mass index (BMI, kg.m-2). When mean and standard deviation were not available, median and interquartile range (IQR) were used, indicated with “*”).

### Primary outcomes

#### Overall mortality

Thirty studies contributed to this analysis, including 12,203 deaths. Each active drug class had direct comparisons with control. The comparison network and forest plots of the direct comparisons are shown in supplementary S3A Fig_Network, S3A Fig_DPP-4_I VERSUS CONTROL, S3A Fig_GLITAZONES VERSUS CONTROL, S3A Fig_GLP-1_A VERSUS CONTROL, S3A Fig_INSULIN VERSUS CONTROL, S3A Fig_METFORMIN VERSUS CONTROL, S3A Fig_SGLT-2_I VERSUS CONTROL, S3A Fig_SULFONYLUREA VERSUS CONTROL, S3A Fig_SULFONYLUREA VERSUS GLITAZONES, S3A Fig_INSULIN VERSUS SULFONYLUREA, S3A Fig_METFORMIN VERSUS SULFONYLUREA. SGLT-2 inhibitors only were associated with a decreased risk of overall mortality compared to control (OR = 0.84 [95% CrI: 0.74; 0.95]) and compared to DPP-4 inhibitors (OR = 0.82 [95% CrI: 0.69; 0.98]). SUCRA values suggested that SGLT-2 inhibitors have the higher probability to be the most efficient treatment (SUCRA = 0.86). SUCRA values for metformin and GLP-1 receptor agonists were relatively similar (0.72 and 0.67, respectively). SUCRA values, summary of the network treatment estimates for each pair of comparisons and for the direct treatment estimates, when available, are summarized in Table 2.

**Table 2:** Treatment effect estimates for overall mortality.

**control ; 0.2**	1.02 (0.89;1.15)	0.93 (0.79;1.09)	0.9 (0.81;1)	0.96 (0.83;1.09)	0.86 (0.67;1.1)	0.84 (0.74;0.95)	0.95 (0.83;1.09)
1.02 (0.89;1.15)	**dpp4_i ; 0.16**	0.91 (0.74;1.12)	0.88 (0.75;1.05)	0.94 (0.78;1.12)	0.84 (0.64;1.12)	0.82 (0.69;0.98)	0.93 (0.77;1.13)
0.88 (0.46;1.39)		**glitazones ; 0.53**	0.97 (0.8;1.18)	1.03 (0.83;1.27)	0.93 (0.69;1.25)	0.91 (0.73;1.11)	1.03 (0.84;1.25)
0.89 (0.81;1)			**glp1_a ; 0.67**	1.07 (0.88;1.25)	0.96 (0.73;1.26)	0.94 (0.79;1.1)	1.06 (0.88;1.26)
0.96 (0.82;1.1)				**insulin ; 0.42**	0.9 (0.68;1.2)	0.88 (0.74;1.06)	0.99 (0.84;1.2)
0.97 (0.63;1.53)					**metformin ; 0.72**	0.98 (0.74;1.3)	1.11 (0.83;1.46)
0.84 (0.67;1.05)						**sglt2_i ; 0.86**	1.13 (0.94;1.37)
0.94 (0.83;1.07)		0.89 (0.56;1.4)		1.01 (0.81;1.25)	2.28 (0.64;8.63)		**sulfonylureas ; 0.44**

The diagonal contains the drug class and its SUCRA value. Treatment effect are OR with its 95% credible interval. Above the diagonal: estimates from the network meta-analysis, OR < 1 is in favor of the column; below the diagonal: estimates from the direct comparison, when available, OR <1 is in favor of the row.

#### Cardiovascular mortality

Twenty-seven studies contributed to the analysis for cardiovascular mortality, including 6,221 cardiovascular deaths. Each active drug class had direct comparisons against control. The comparison network and forest plots of the direct comparisons are shown in supplementary S3B Fig_Network, S3B Fig_DPP-4_I VERSUS CONTROL, S3B Fig_GLITAZONES VERSUS CONTROL, S3B Fig_GLP-1_A VERSUS CONTROL, S3B Fig_INSULIN VERSUS CONTROL, S3B Fig_METFORMIN VERSUS CONTROL, S3B Fig_SGLT-2_I VERSUS CONTROL, S3B Fig_SULFONYLUREA VERSUS CONTROL, S3B Fig_SULFONYLUREA VERSUS GLITAZONES, S3B Fig_INSULIN VERSUS SULFONYLUREA, S3B Fig_METFORMIN VERSUS SULFONYLUREA. No significant differences were observed in the network comparisons. SUCRA values suggested SGLT-2 inhibitors have the higher probability to be the most efficient treatment (SUCRA = 0.8), followed by GLP-1 receptor agonists and metformin (0.63 and 0.55, respectively). SUCRA values, network and direct comparisons are summarized in Table 3.

**Table 3:** Treatment effect estimates for cardiovascular mortality.

**control ; 0.24**	0.99 (0.8;1.19)	0.92 (0.71;1.21)	0.89 (0.76;1.05)	0.95 (0.7;1.3)	0.91 (0.65;1.27)	0.83 (0.69;1)	0.92 (0.74;1.16)
0.99 (0.85;1.14)	**dpp4_i ; 0.31**	0.94 (0.68;1.32)	0.9 (0.71;1.18)	0.96 (0.68;1.4)	0.92 (0.63;1.36)	0.84 (0.65;1.11)	0.93 (0.7;1.28)
0.91 (0.4;2.07)		**glitazones ; 0.51**	0.96 (0.71;1.31)	1.03 (0.69;1.53)	0.98 (0.64;1.5)	0.9 (0.64;1.23)	0.99 (0.71;1.39)
0.89 (0.78;1.02)			**glp1_a ; 0.63**	1.07 (0.76;1.5)	1.02 (0.7;1.47)	0.94 (0.72;1.18)	1.03 (0.78;1.37)
0.93 (0.73;1.19)				**insulin ; 0.43**	0.95 (0.61;1.49)	0.87 (0.61;1.24)	0.96 (0.7;1.34)
1.15 (0.5;3)					**metformin ; 0.55**	0.92 (0.62;1.34)	1.01 (0.69;1.5)
0.83 (0.61;1.12)						**sglt2_i ;** **0.8**	1.1 (0.83;1.5)
0.9 (0.75;1.08)		0.79 (0.27;2.15)		0.76 (0.61;0.95)	1.72 (0.54;5.52)		**Sulfonylureas ; 0.53**

The diagonal contains the drug class and its SUCRA value. Treatment effect are OR with its 95% credible interval. Above the diagonal: estimates from the network meta-analysis, OR < 1 is in favor of the column; below the diagonal: estimates from the direct comparison, when available, OR <1 is in favor of the row.

#### Major adverse cardiovascular events (MACE)

Twenty-seven studies contributed to the analysis for MACE, including 17,188 MACEs. Details regarding the number of events are presented in S5 Table. Each active drug class had direct comparisons against control. The comparison network and forest plot of the direct comparisons are shown in supplementary S3C Fig_Network, S3C Fig_DPP-4_I VERSUS CONTROL, S3C Fig_GLITAZONES VERSUS CONTROL, S3C Fig_GLP-1_A VERSUS CONTROL, S3C Fig_INSULIN VERSUS CONTROL, S3C Fig_METFORMIN VERSUS CONTROL, S3C Fig_SGLT-2_I VERSUS CONTROL, S3C Fig_SULFONYLUREA VERSUS CONTROL, S3C Fig_SULFONYLUREA VERSUS GLITAZONES, S3C Fig_INSULIN VERSUS SULFONYLUREA, S3C Fig_METFORMIN VERSUS SULFONYLUREA. Compared to control, only SGLT-2 inhibitors and GLP-1 receptor agonists were associated with a decreased risk of MACE (OR = 0.89 [95% CrI: 0.81; 0.98] and OR = 0.88 [95% CrI: 0.81; 0.95], respectively). Compared to DPP-4 inhibitors, only GLP-1 receptor agonists were associated with a decreased risk of MACE (OR = 0.88 [95% CrI: 0.79; 0.99]). Insulin was also associated with an increased risk of MACE compared to GLP-1 receptor agonists (OR = 1.19 [95% CrI: 1.01; 1.42]). SUCRA values suggested GLP-1 receptor agonists have the higher probability to be the most efficient treatment (SUCRA = 0.76), followed by metformin and SGLT-2 inhibitors (SUCRA values: 0.75 and 0.71, respectively). SUCRA values, network and direct comparisons are summarized in Table 4. Ranking probability curve for MACE is presented in the supplementary S4 Fig.

**Table 4:** Treatment effect estimates for major adverse cardiovascular events (MACE).

**control ; 0.21**	0.99 (0.91;1.08)	0.9 (0.79;1.03)	0.88 (0.81;0.95)	1.05 (0.9;1.21)	0.85 (0.65;1.11)	0.89 (0.81;0.98)	0.93 (0.81;1.06)
0.98 (0.92;1.05)	**dpp4_i ; 0.26**	0.9 (0.78;1.07)	0.88 (0.79;0.99)	1.05 (0.89;1.25)	0.85 (0.65;1.14)	0.9 (0.79;1.02)	0.94 (0.8;1.1)
0.86 (0.58;1.17)		**glitazones ; 0.66**	0.98 (0.83;1.14)	1.17 (0.95;1.42)	0.94 (0.71;1.27)	0.99 (0.83;1.16)	1.04 (0.87;1.22)
0.87 (0.74;1)			**glp1_a ; 0.76**	1.19 (1.01;1.42)	0.97 (0.73;1.29)	1.01 (0.9;1.15)	1.06 (0.91;1.24)
1.04 (0.93;1.17)				**insulin ; 0.12**	0.81 (0.6;1.11)	0.85 (0.71;1.01)	0.89 (0.72;1.09)
0.97 (0.65;1.44)					**metformin ; 0.75**	1.05 (0.79;1.39)	1.09 (0.82;1.44)
0.89 (0.79;0.99)						**sglt2_i ; 0.71**	1.04 (0.89;1.23)
0.93 (0.81;1.08)		0.87 (0.54;1.3)			1.62 (0.77;3.47)		**sulfonylureas ; 0.53**

The diagonal contains the drug class and its SUCRA value. Treatment effect are OR with its 95% credible interval. Above the diagonal: estimates from the network meta-analysis, OR < 1 is in favor of the column; below the diagonal: estimates from the direct comparison, when available, OR <1 is in favor of the row.

### Secondary outcomes

Regarding the risk of MI, metformin was almost associated with a decreased risk of non-fatal MI compared to control (OR = 0.66 [95% CrI: 0.44; 1]). Regarding the risk of stroke, glitazones were associated with a decreased risk of all strokes compared to control and DPP-4 inhibitors (OR = 0.74 [95% CrI: 0.57; 0.95] and OR = 0.72 [95% CrI: 0.52; 0.98], respectively); sulfonylureas and SGLT-2 inhibitors were associated with an increased risk of stroke compared to glitazones (OR = 1.53 [95% CrI: 1.13; 2.15] and OR = 1.45 [95% CrI: 1.06; 2.03], respectively). Insulin was associated with an increased risk of severe adverse events compared to all the comparison except the sulfonylureas: increased risk with insulin compared to control, DPP-4 inhibitors, glitazones, GLP-1 receptor agonists (OR = 1.32 [95% CrI: 1.05; 1.68], OR = 1.44 [95% CrI: 1.05; 1.97], OR = 1.37 [95% CrI: 1.04; 1.81], OR = 1.43 [95% CrI: 1.11; 1.85], respectively), decreased risk with metformin and SGLT-2 inhibitors compared to insulin (OR = 0.7 [95% CrI: 0.5; 0.99] and OR = 0.67 [95% CrI: 0.51; 0.87], respectively). Insulin and sulfonylureas both were associated with an increased risk of severe hypoglycemia compared to all the other comparison except the metformin: i) increased risk with insulin compared to control, DPP-4 inhibitors, glitazones, GLP-1 receptor agonists (OR = 3.44 [95% CrI: 1.76; 7.25], OR = 2.92 [95% CrI: 1.22; 7.64], OR = 2.99 [95% CrI: 1.17; 7.97], OR = 4.14 [95% CrI: 1.95; 10.13], respectively), ii) decreased risk with SGLT-2 inhibitors compared to insulin (OR = 0.23 [95% CrI: 0.08; 0.59]), iii) increased risk with sulfonylureas compared to control, DPP-4 inhibitors, glitazones, GLP-1 receptor agonists and SGLT-2 inhibitors (OR = 2.9 [95% CrI: 1.68; 6.25], OR = 2.45 [95% CrI: 1.18; 6.61], OR = 2.52 [95% CrI: 1.22; 6.32], OR = 3.49 [95% CrI: 1.82; 8.94], OR = 3.71 [95% CrI: 1.62; 11] respectively). For secondary outcomes, treatment effect estimates against control are summarized in Table 5.

**Table 5:** Summary of treatment effect compared to control for secondary outcomes.

	All.MI	Non.fatal.MI	All.Stroke	Non.fatal.Stroke	SAE	Sev.Hypo
dpp4_i	0.95 (0.78;1.15)	1.01 (0.85;1.2)	1.03 (0.85;1.26)	0.92 (0.69;1.21)	0.92 (0.75;1.12)	1.18 (0.67;2.06)
glitazones	1.18 (0.78;1.78)	0.91 (0.74;1.11)	0.74 (0.57;0.95)	0.78 (0.55;1.11)	0.97 (0.83;1.12)	1.15 (0.58;2.33)
glp1_a	0.91 (0.79;1.02)	0.94 (0.83;1.05)	0.89 (0.77;1.04)	0.88 (0.73;1.06)	0.93 (0.85;1.01)	0.83 (0.52;1.23)
insulin	0.98 (0.79;1.2)	0.95 (0.68;1.31)	0.99 (0.8;1.19)	0.71 (0.43;1.14)	1.32 (1.05;1.68)	3.44 (1.76;7.25)
metformin	0.8 (0.6;1.1)	0.66 (0.44;1)	0.73 (0.46;1.1)	0.62 (0.36;1.03)	0.93 (0.73;1.19)	1.34 (0.31;5.63)
sglt2_i	0.88 (0.72;1.07)	0.87 (0.73;1.04)	1.07 (0.88;1.31)	1.04 (0.82;1.31)	0.88 (0.77;1.01)	0.78 (0.39;1.55)
sulfonylureas	0.87 (0.65;1.16)	0.93 (0.76;1.13)	1.13 (0.95;1.39)	1.02 (0.8;1.28)	1.03 (0.86;1.17)	2.9 (1.68;6.25)

MI: myocardial infarction; SAE: serious adverse events; Sev.hypo: severe hypoglycemia; “_i” stands for inhibitor, “_a” stands for agonist.

### Statistical assessment

Convergences were reached for all the analyses. Residual deviance was globally acceptable (for overall mortality, ratio of Dbar/number of data points was 1.074). Heterogeneity of the treatment effect was globally low (I^2^ for overall mortality: 8%). Network consistency was globally satisfying. For overall mortality, the network estimation of metformin against sulfonylurea was inconsistent with the direct comparison (see discussion).

### Sensitivity analyses

When restricting the analysis to double-blinded studies only, the decreased risk of overall mortality with SGLT-2 and of MACE with SGLT-2 and GLP-1 agonist remained, but treatment estimation were not interpretable for metformin and sulfonylureas due to inconsistency.

The mean prevalence of previous cardiovascular history at baseline across all trials was 54.9% +/- 29.4 (see supplementary S4 Table). There were 17 trials in the subgroup with a majority of patient with previous CV history (‘high CV risk’ subgroup), and 16 trials in the subgroup with a majority of patient without previous CV history (‘low CV risk’ subgroup). There was no trial comparing metformin to control in the ‘high CV risk’ subgroup. There were no GLP-1 receptor agonist trials and no DPP-4 inhibitor trials in the ‘low CV risk’ subgroup. The beneficial effects of SGLT-2 inhibitors and of GLP-1 receptor agonists remained in the ‘high CV risk’ subgroup. In the trials including a majority of patients without previous CV history, the comparisons of SGLT-2 inhibitors, metformin and control did not showed significant differences on primary outcomes. Compared to control, risk of overall mortality, CV mortality and of MACE, with SGLT-2 inhibitors, was: OR = 0.92 [95% CrI: 0.55; 1.57], OR = 0.99 [95% CrI: 0.29; 3.28], OR = 0.94 [95% CrI: 0.55; 1.6], respectively. Compared to control, risk of overall mortality, CV mortality and of MACE, with metformin, was: OR = 0.94 [95% CrI: 0.67; 1.41], OR = 1.08 [95% CrI: 0.57; 2.43], OR = 0.97 [95% CrI: 0.57; 1.56], respectively. Compared to metformin, risk of overall mortality, of CV mortality and of MACE, with SGLT-2 inhibitors was: OR = 0.99 [95% CrI: 0.5; 1.78], OR = 0.93 [95% CrI: 0.21; 3.29], OR = 0.96 [95% CrI: 0.48; 2.09], respectively.

The mean difference of HbA1c during the follow up was -0.43% +/- 0.22. Available data for defining the glycemic contrast was unfortunately heterogeneous between studies, limiting the exploration of this potential effect modifier.

## Discussion

### Main findings

Our study confirms the beneficial effects of SGLT-2 inhibitors and GLP-1 receptor agonist on MACEs with at least two positive independent trials. SGLT-2 inhibitors only were associated with a decreased risk of overall mortality compared to control and to DPP-4 inhibitors. GLP-1 agonists were only associated with a decreased risk of major adverse cardiovascular events compared to control, DPP-4 inhibitors and insulin. Metformin did not showed any benefits on mortality or major adverse cardiovascular events. Glitazones were associated with a decreased risk of stroke, insulin with an increased risk of serious adverse events, insulin and sulfonylureas with an increased risk of severe hypoglycemia. In the subgroup of trials including a majority of patients without previous cardiovascular history, the comparisons of SGLT-2 inhibitors, metformin and control did not showed significant differences on those outcomes. This subgroup did not include trials assessing GLP-1 agonists.

### Strengths of the study

Several hypoglycemic drug classes are now available. However, only a few direct comparisons between active treatments are available. New hypoglycemic drug classes especially have been compared only to placebo for their effect on cardiovascular outcomes. Thus, in order to compare all the hypoglycemic drug classes, network meta-analyses are needed for taking into account the information from both direct and indirect comparisons. We included any hypoglycemic drug classes, old or new, whose have been assessed for major cardiovascular outcomes, for the first time in the same network meta-analysis. We also included the last powerful trials. Moreover, we conducted subgroup analyses according to the prevalence of previous CV history in each trials. Our study helps to summarize the results of clinical trials in type 2 diabetes, focusing on major cardiovascular outcomes. Regarding the SGLT-2 inhibitors, the decrease in overall mortality with SGLT-2 inhibitors is mainly driven by the EMPAREG OUTCOME trial [2]. Moreover, a potential warning signal has been observed for peripheral amputations [48]. The CANVAS Program was the pooling of the CANVAS trial and the CANVAS R trial [49]. Those trials were initially planned separately. After an unplanned interim analysis of the CANVAS trial, those two trials have been joined together to increase the power, both trials having very similar design and inclusion criteria. This has been well explicated and justified before the publication of the final results [49]. However, the results regarding overall and cardiovascular mortality are presented on the full dataset, including data which have been used for the interim analysis. Surprisingly, the effect of GLP-1 receptor agonists was no more significant for overall mortality, with the recently published HARMONY OUTCOME trial [8]. Regarding the other classes, the effect of metformin was consistent with previous meta-analyses [47]. The beneficial effect of glitazones regarding the risk of stroke has already been described [50]. The neutral effect of DPP-4 inhibitors on cardiovascular events was consistent with previous meta-analyses [51]. The increased risk of severe hypoglycemia with sulfonylureas and insulin was also consistent with their mechanism of action and previous knowledge [52]. The increased risk of severe adverse event with insulin is based on the data of ORIGIN [36]; we found neither severe adverse event data for UGDP [53] nor UKPDS 33 [24]. Therefore our results mostly reflect the increased risk of hypoglycemia as described in the ORIGIN trial. We did not assess the specific risk of cardiac insufficiency. Unfortunately, we did not find any studies evaluating alpha glucosidase inhibitors or glinides on such major clinical outcome.

Previous network meta-analyses [54–57] did not include both the old and the new hypoglycemic drug classes and the last powerful trials (EXSCEL [45], HARMONY OUTCOME [8], DECLARE TIMI 58 [9] and CARMELINA [22]). Above all, our results differ slightly from the network meta-analysis of Zheng et al [6], as the GLP-1 agonist were no more associated with a decrease in overall mortality, due to the latest HARMONY OUTCOME trial. Moreover, our results challenge the recently suggested benefit of SGLT-2 inhibitors in primary cardiovascular prevention [10], as we did not showed a significant effect of SGLT-2 inhibitors compared to control and metformin on major CV outcomes, in trials including a majority of patients without previous CV history.

### Limitations

Our study has some limitations. We included double-blinded and open clinical trials, which lead to a risk of bias. Unfortunately, there were many open trials in T2D in the last decades. However, the sensitivity analysis restricted to the double-blinded studies was consistent with the main results. We included trials from 1970 to 2018. New hypoglycemic drugs were not assessed in the same medical context as old hypoglycemic drugs. The research of glycemic equipoise between arms in the recent trials could interfere with the interpretation of the results. Old hypoglycemic drugs have also been evaluated mostly in subjects with a shorter duration of type 2 diabetes, while the complications occur after several years of hyperglycemia. We limited our analysis on macroangiopathy. They are not the only complications of T2D patients, but they are the main cause of death in this population. We planned to address microvascular complications, but their reporting was not homogenous enough to allow the analysis. We also limited our analysis at the therapeutic class level. Treatment effect heterogeneity within classes has been described notably for glitazones and sulfonylureas, and our analysis could hide specific molecular effects by averaging the drug class effect. Pooling rosiglitazone and pioglitazone trials could have hidden some beneficial effect of pioglitazone [58] because of the negative cardiovascular effects of rosiglitazone [59]. Moreover, the sulfonylureas were mainly studied through the ADVANCE study [30], which compared a specific sulfonylurea against active hypoglycemic drugs including other sulfonylureas. Unfortunetaly, available data did not allow assessing the specific molecular effect. We planned to use the SUCRA values for ranking the drugs, but it does not take into consideration the upper bound of the credible interval. Thus it resulted for example in a better ranking of Metformin compared to SGLT-2 inhibitors for MACE, whereas metformin’s effect was not significant. Slight inconsistency has been observed for metformin. This seems to be due to the direct comparison of metformin against sulfonylureas in the SPREAD DIMCAD trial [38]. Moreover, some drug classes have not been studied in certain populations (no DPP-4 inhibitors or GLP-1 agonist trials in the ‘low CV risk’ subgroup), which limit the assessment of the transitivity assumption. Sensitivity analysis did not allow identification of glycemic contrast during the trials as potential effect modifiers. Previous meta-regression looking for an association between HbA1c decrease and clinical events showed conflicting results [60, 61]. Unfortunately, the reporting of the glycemic exposure in included trials was not well standardized, and the available data were heterogeneous.

### Implications

SGLT-2 inhibitors and GLP-1 receptor agonists are recommanded for patients with clinical cardiovascular disease [7]. It has been recently suggested that SGLT-2 inhibitors could also be helpful in primary CV prevention [10], but metformin remained the first-line therapy for glucose lowering medication in the last international guidelines [7]. Our study challenges the suggested benefit of SGLT-2 inhibitors in primary cardiovascular prevention, as we did not observe significant difference on overall mortality or MACE between SGLT-2 inhibitors, metformin and control. Thus, our results showed the need for direct comparisons of SGLT-2 inhibitors, GLP-1 agonists and metformin, notably in primary cardiovascular prevention. Moreover, integration in network meta-analysis of supplementary active direct comparisons as the CAROLINA trial [62] will be helpful to better compare the hypoglycemic drugs. Integration of other comparison of SGLT-2 inhibitors to placebo as the VERTIS CV trials [63] will also be helpful, as the effect of SGLT-2 on mortality is mostly driven by the EMPAREG-OUTCOME, and as the decrease in overall mortality with GLP-1 agonist was no more significant with the HARMONY OUTCOMES trial. Likewise, further meta-analyses are needed for assessing the relative effect of glucose lowering drugs on microangiopathy, and for assessing the heterogeneity in the treatment effect within therapeutic classes. Finally, it would be interesting to model the cost efficiency of hypoglycemic drugs with those treatment effect estimations.

## Conclusion

Hypoglycemic drugs are used to control glycaemia and reduce diabetic complications in tens of millions of people worldwide. This study helps to summarize factual knowledge of those therapeutic classes on major clinical outcomes. SGLT-2 inhibitors and GLP-1 receptor agonists appear to have the most beneficial effects on MACE, especially in type 2 diabetic patients with previous cardiovascular diseases.

## Supporting information

S1 Fig PRISMA network meta-analysis checklist of GLUCOSE DINET. (DOCX)

S2 Fig **Risk of bias assessment (A: summary, B: details)**.
(DOCX)

S3 Fig Network and forest plot for the primary outcomes. (DOCX)

S4 Fig Probability curves of each drug classes to be ranked best treatment to the last effective for major adverse cardiovascular events. (DOCX)

S1 Table Search strategy used for medline. (DOCX)

S2 Table Definitions of the major adverse cardiovascular events (MACE) outcome for each trial. (DOCX)

S3 Table Reported definitions of the serious adverse event (SAE) outcome used for each trial. (DOCX)

S4 Table Baseline cardiovascular risk groups for the sensitivity analysis. (DOCX)

S5 Table Dataset GLUCOSE DINET. (XLSX)

## Abbreviations

BMI: Body mass index
CV: Cardiovascular
CVD: Cardiovascular diseases
FDA: Food and Drug Administration
HbA1c: Glycated hemoglobin
MACE: Major Adverse Cardiovascular Event
OR: Odds ratio
RCTs: Randomized clinical trials
SUCRA: Surface Under the Cumulative Ranking Curve
T2D: Type 2 Diabetes
