# Supplementary material for: GLUcose COntrol Safety & Efficacy in type 2 DIabetes, a systematic review and NETwork meta-analysis
Source: PLoS One. 2019 Jun 25;14(6):e0217701. doi: 10.1371/journal.pone.0217701 (PMC6592598; doi:10.1371/journal.pone.0217701)
Supplement: S2 Fig — Risk of bias assessment (A: summary, B: details). (DOCX) [file pone.0217701.s002.docx]

**S2 Appendix Risk of bias assessment (A: summary, B: details)**

 A. Summary of risk of bias assessment.


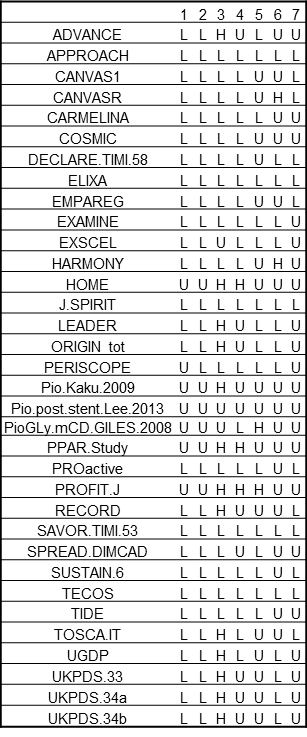


B. Risk of bias assessment for each included study

(L: low risk of bias, U: unclear, H: high. 1: Sequence generation, 2: Allocation concealment, 3: Blinding of participants, 4: Blind-ing of outcome, 5: Attrition bias, 6: Reporting bias, 7: Other risk of bias)
