# Supplementary material for: GLUcose COntrol Safety & Efficacy in type 2 DIabetes, a systematic review and NETwork meta-analysis
Source: PLoS One. 2019 Jun 25;14(6):e0217701. doi: 10.1371/journal.pone.0217701 (PMC6592598; doi:10.1371/journal.pone.0217701)
Supplement: S3 Fig — (DOCX) [file pone.0217701.s003.docx]

**S3 Fig. Network figures for the primary outcomes and forest plots of the direct comparisons**

1. **Overall mortality**

This network included 30 randomized trials. On 168 332 subjects, 12 203 presented the outcome.

S3A Fig_Network:

Forest plots of the overall mortality for each direct comparison:

S3A Fig_DPP-4_I VERSUS CONTROL


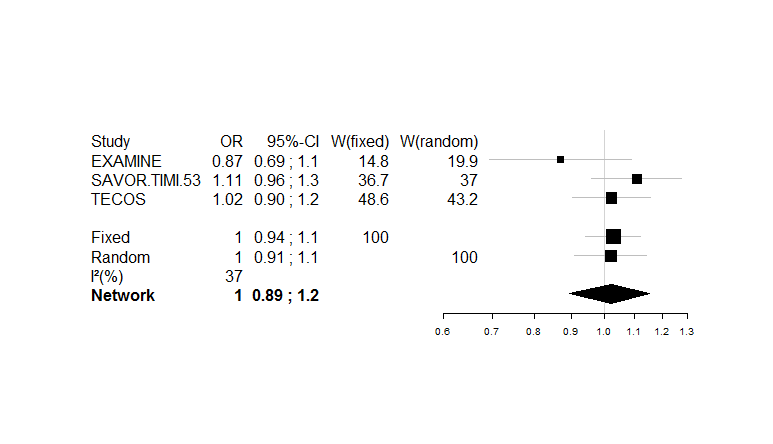


S3A Fig_GLITAZONES VERSUS CONTROL


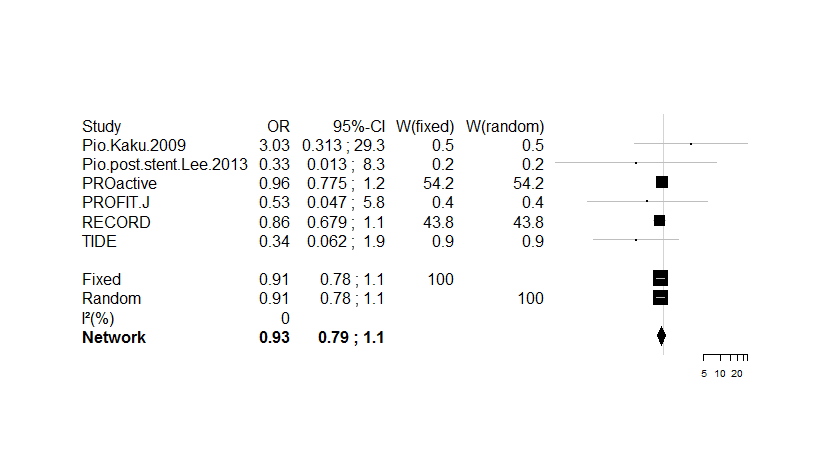


S3A Fig_GLP-1_A VERSUS CONTROL


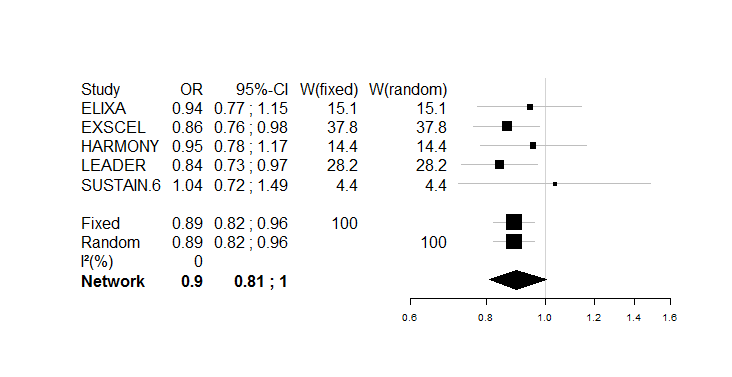


S3A Fig_INSULIN VERSUS CONTROL


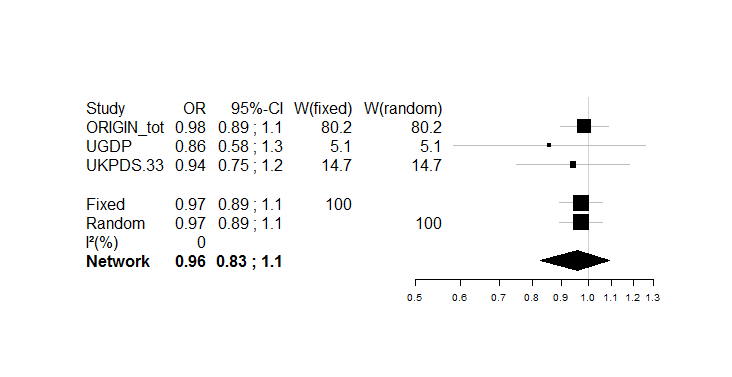


S3A Fig_METFORMIN VERSUS CONTROL


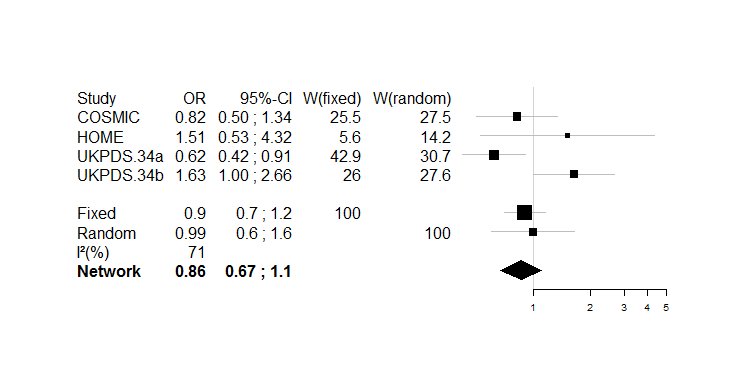


S3A Fig_SGLT-2_I VERSUS CONTROL


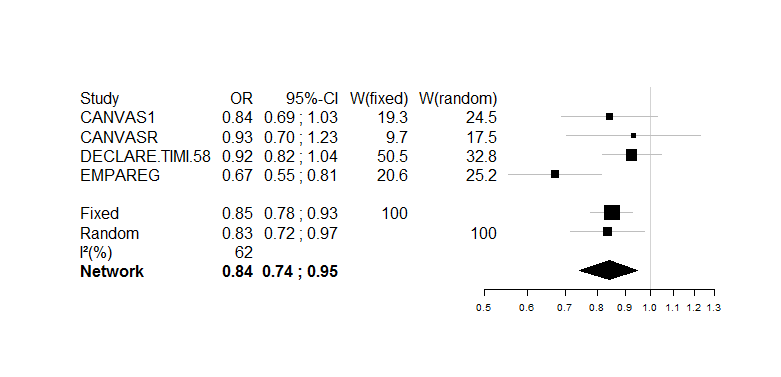


S3A Fig_SULFONYLUREA VERSUS CONTROL


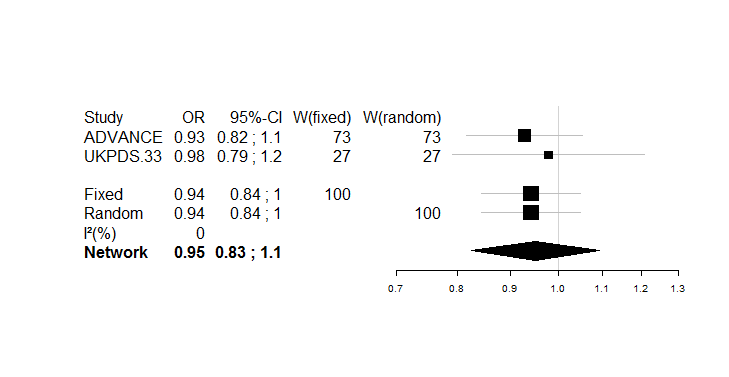


S3A Fig_SULFONYLUREA VERSUS GLITAZONES


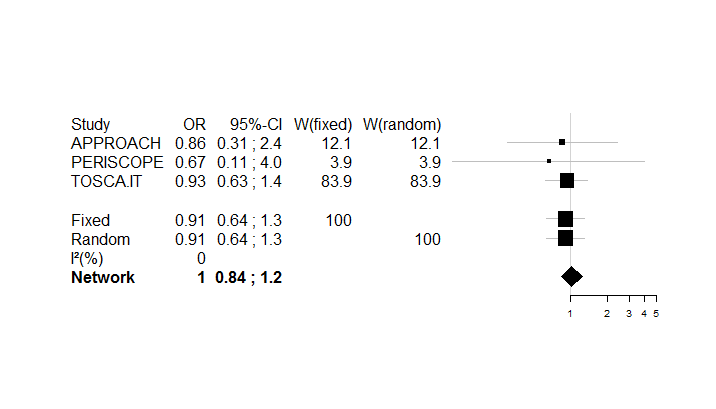


S3A Fig_INSULIN VERSUS SULFONYLUREA

Not applicable (one study only)

S3A Fig_METFORMIN VERSUS SULFONYLUREA

Not applicable (one study only)

1. **Cardiovascular mortality**

This network included 27 randomized trials. On 152 642 subjects, 6 221 presented the outcome.

S3B Fig_Network:

Forest plots of the cardiovascular mortality for each direct comparison:

S3B Fig_DPP-4_I VERSUS CONTROL


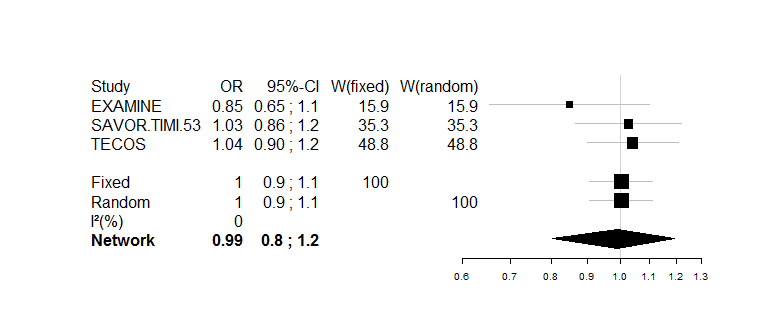


S3B Fig_GLITAZONES VERSUS CONTROL


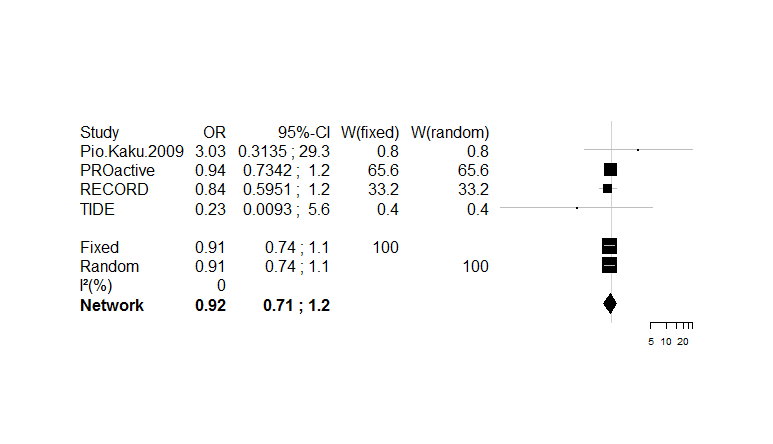


S3B Fig_GLP-1_A VERSUS CONTROL


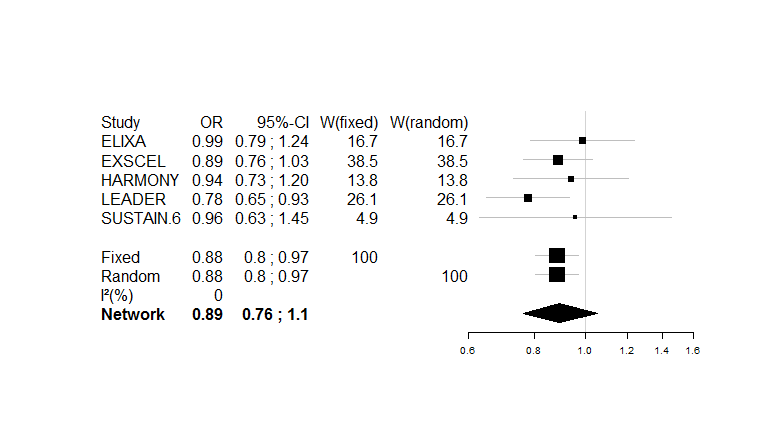


S3B Fig_INSULIN VERSUS CONTROL


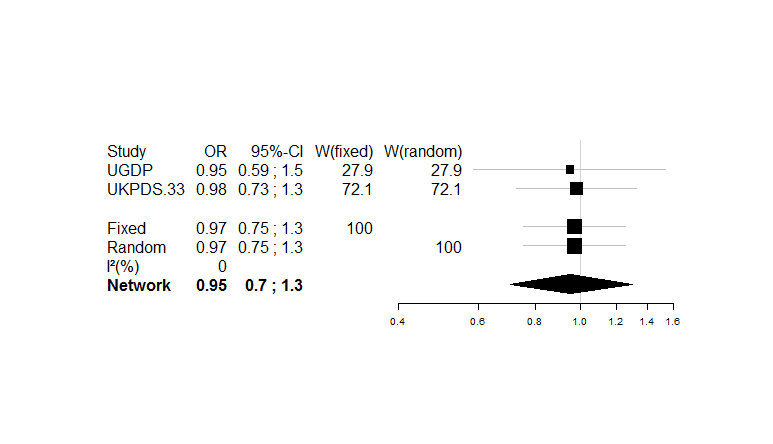


S3B Fig_METFORMIN VERSUS CONTROL


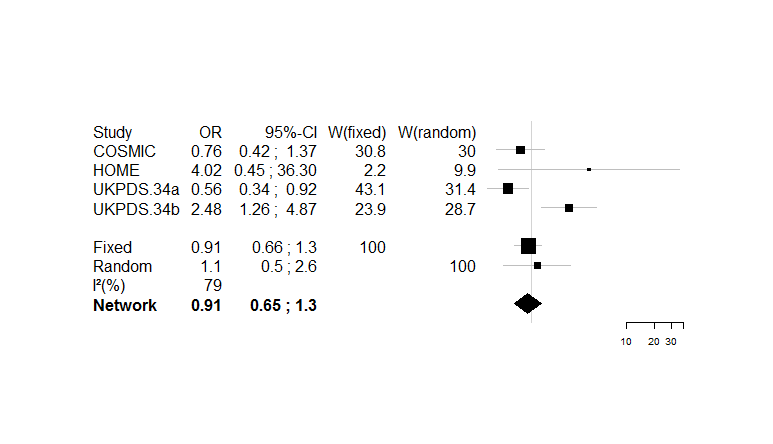


S3B Fig_SGLT-2_I VERSUS CONTROL


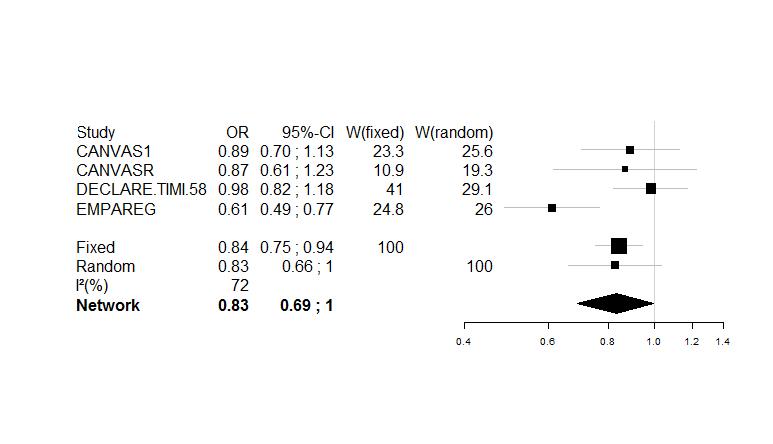


S3B Fig_SULFONYLUREA VERSUS CONTROL


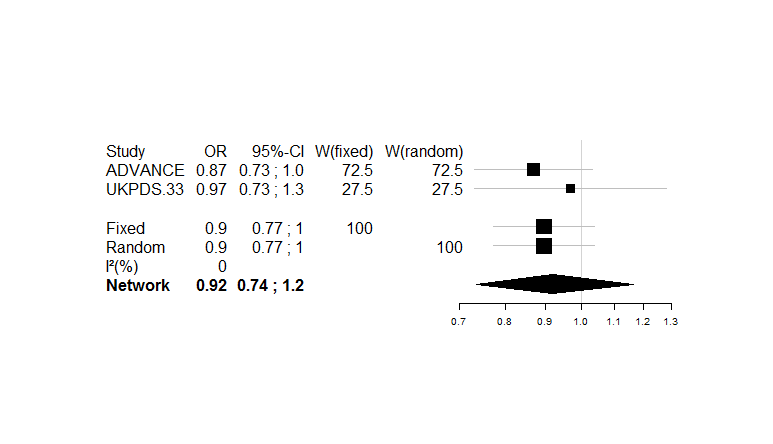


S3B Fig_SULFONYLUREA VERSUS GLITAZONES


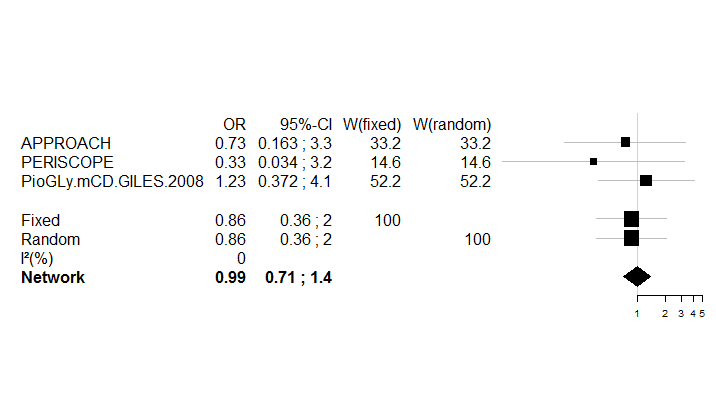


S3B Fig_INSULIN VERSUS SULFONYLUREA

Not applicable (one study only)

S3B Fig_METFORMIN VERSUS SULFONYLUREA

Not applicable (one study only)

1. **Major Adverse Cardiovascular Events (MACE)**

This network included 27 randomized trials. On 168 068 subjects, 17 188 presented the outcome.

S3C Fig_Network:

Forest plots of the Major Adverse Cardiovascular Events (MACE) for each direct comparison:

S3C Fig_DPP-4_I VERSUS CONTROL


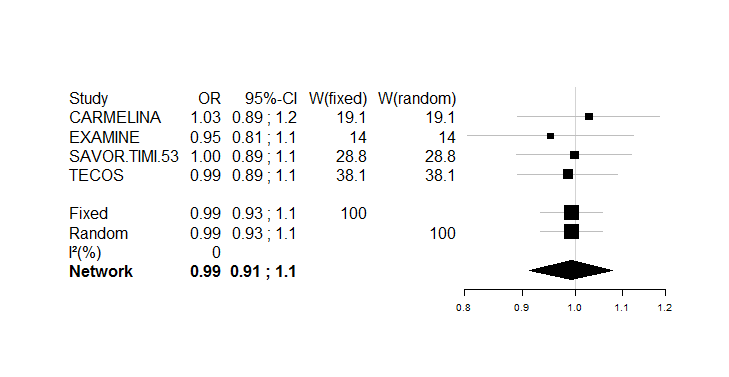


S3C Fig_GLITAZONES VERSUS CONTROL


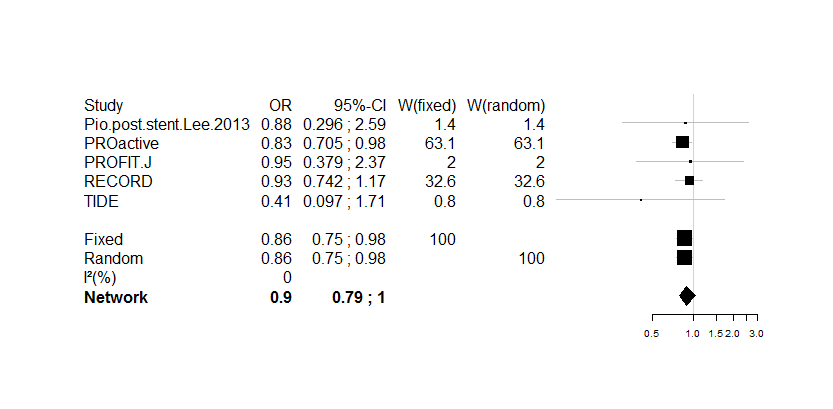


S3C Fig_GLP-1_A VERSUS CONTROL


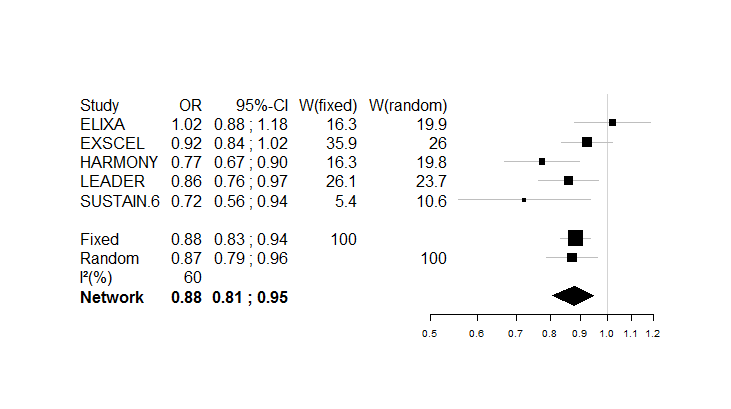


S3C Fig_INSULIN VERSUS CONTROL


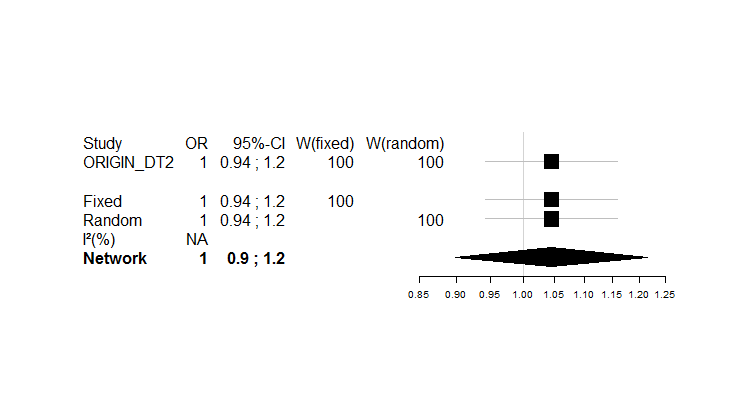


S3C Fig_METFORMIN VERSUS CONTROL


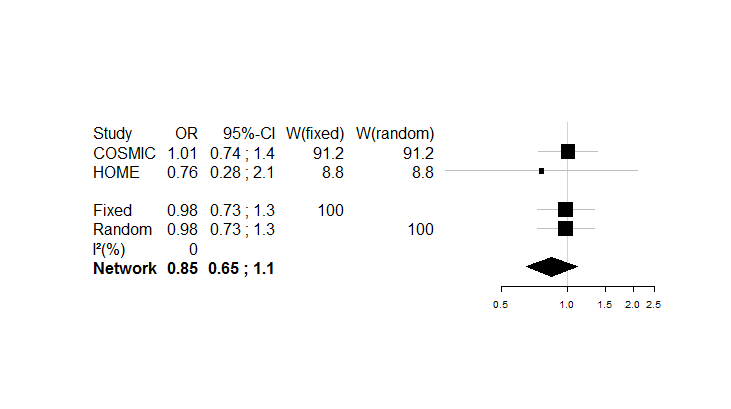


S3C Fig_SGLT-2_I VERSUS CONTROL


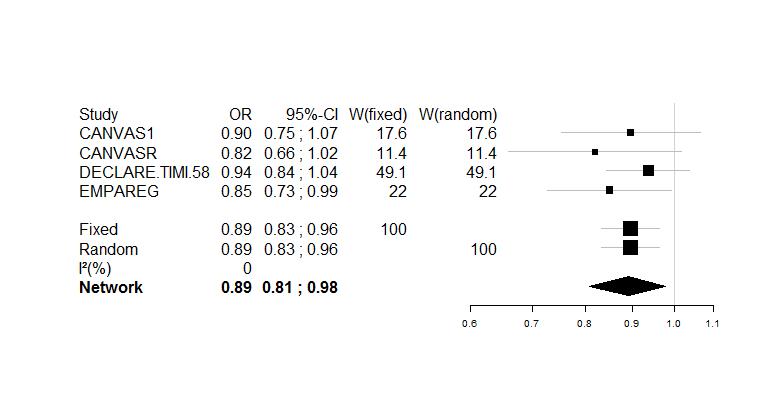


S3C Fig_SULFONYLUREA VERSUS CONTROL


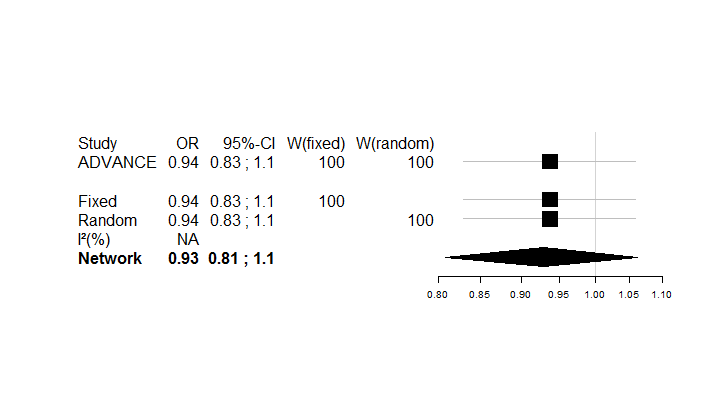


S3C Fig_SULFONYLUREA VERSUS GLITAZONES


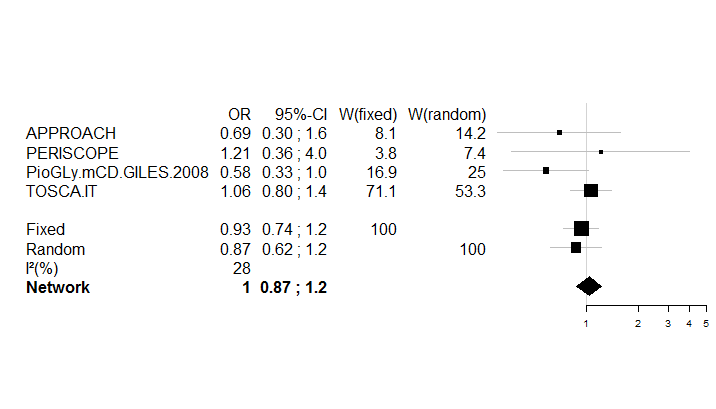


S3C Fig_INSULIN VERSUS SULFONYLUREA

Not applicable (one study only)

S3C Fig_METFORMIN VERSUS SULFONYLUREA

Not applicable (one study only)
