# Supplementary material for: GLUcose COntrol Safety & Efficacy in type 2 DIabetes, a systematic review and NETwork meta-analysis
Source: PLoS One. 2019 Jun 25;14(6):e0217701. doi: 10.1371/journal.pone.0217701 (PMC6592598; doi:10.1371/journal.pone.0217701)
Supplement: S4 Fig — (DOCX) [file pone.0217701.s004.docx]

**S4 Appendix. Probability curves of each drug classes to be ranked best treatment to the last effective for major adverse cardiovascular events**

Probability curves of each drug classes to be ranked best treatment (rank = 1) to the last effective (rank = 8), for major adverse cardiovascular events (MACE).
