# Supplementary material for: GLUcose COntrol Safety & Efficacy in type 2 DIabetes, a systematic review and NETwork meta-analysis
Source: PLoS One. 2019 Jun 25;14(6):e0217701. doi: 10.1371/journal.pone.0217701 (PMC6592598; doi:10.1371/journal.pone.0217701)
Supplement: S1 Table — (DOCX) [file pone.0217701.s005.docx]

| Patients | "diabetes mellitus, type 2"[MeSH Terms]  OR type 2 diabetes mellitus[Title/Abstract]  OR type 2 diabetes[Title/Abstract]  OR "diabetes mellitus"[MeSH Terms]  OR "diabetes mellitus"[Title/Abstract]  OR "non-insulin-dependent diabetes mellitus"[Title/Abstract]  OR "non insulin dependent diabetes mellitus"[Title/Abstract]  OR "NIDDM"[Title/Abstract] |
| --- | --- |
|  | AND |
| Intervention | hypoglycaemic agents[Title/Abstract]  OR hypoglycemic agents[Pharmacological Action]  OR "hypoglycemic agents"[MeSH Terms]  OR "hypoglycemic agents"[Title/Abstract]  OR "hypoglycemic agent"[Title/Abstract]  OR "glycaemic control"[Title/Abstract]  OR "glycemic control"[Title/Abstract]  OR "glucose control"[Title/Abstract] |
|  | AND |
| Type of study | (randomized controlled trial[pt]  OR controlled clinical trial[pt]  OR randomized[tiab]  OR placebo[tiab]  OR "clinical trials as topic"[MeSH Terms:noexp]  OR randomly[tiab]  OR trial[ti])  NOT (animals[mh] NOT humans[mh]) |
|  | AND |
| Outcome | "mortality"[Subheading]  OR mortality[Title/Abstract]  OR "mortality"[MeSH Terms]  OR "cardiovascular diseases"[MeSH Terms]  OR "cardiovascular diseases"[Title/Abstract]  OR "cardiovascular disease"[Title/Abstract] |
|  | NOT |
| Type of studies excluded | review[Publication Type]  OR meta analysis[Publication Type]  OR meta-analysis[Publication Type] |

**S1 Table. Search strategy used for Medline**
