# Supplementary material for: GLUcose COntrol Safety & Efficacy in type 2 DIabetes, a systematic review and NETwork meta-analysis
Source: PLoS One. 2019 Jun 25;14(6):e0217701. doi: 10.1371/journal.pone.0217701 (PMC6592598; doi:10.1371/journal.pone.0217701)
Supplement: S2 Table — (DOCX) [file pone.0217701.s006.docx]

**S2 Table. Definitions of the major adverse cardiovascular events (MACE) outcome for each trial**. When not available, proxy was used as defined in the table. CV: cardiovascular, MI: myocardial infarction, SAE: serious adverse event, PCI: percutaneous coronary intervention, HF: heart failure

| **Study** | **"MACE" outcome** | **"MACE" outcome reported definitions** |
| --- | --- | --- |
| ADVANCE | MACE | CV death and non-fatal MI and non-fatal stroke |
| APPROACH | MACE | CV death, non-fatal MI, or non-fatal stroke |
| CANVAS | MACE | CV mortality, non-fatal MI, non- fatal stroke |
| CANVASR | MACE | CV mortality, non-fatal MI, non- fatal stroke |
| CARMELINA | MACE | CV mortality, non-fatal MI, non- fatal stroke |
| COSMIC | proxy | SAE Cardiac disorders, including coronary artery disease, chest pain, congestive cardiac failure, myocardial infarction +/- cerebrovascular accident? |
| DECLARE TIMI 58 | MACE | CV death, myocardial infarction, or ischemic stroke |
| ELIXA | proxy | Death from CV causes, non-fatal stroke, non-fatal MI, or unstable angina |
| EMPAREG | MACE | Death from CV causes, non-fatal MI, or non-fatal stroke |
| EXAMINE | MACE | Death from CV causes, non-fatal MI, or non-fatal stroke |
| EXSCEL | MACE | Death from CV causes, non-fatal MI, or non-fatal stroke |
| HARMONY | MACE | Death from CV causes, non-fatal MI, or non-fatal stroke |
| HOME | proxy | CV intervention (peripheral arterial reconstruction, percutaneous transluminal coronary angioplasty, and coronary artery bypass graft) |
| J-SPIRIT | NA |  |
| LEADER | MACE | Death from CV causes, non-fatal (including silent) MI, or non-fatal stroke |
| ORIGIN | MACE | CV death, non-fatal MI, non-fatal stroke |
| PERISCOPE | MACE | CV death, non-fatal MI, or non-fatal stroke |
| Kaku.2009 | proxy | Death, acute MI excluding silent MI, or stroke |
| Lee.2013 | proxy | Death, MI, re-PCI TLR (target lesion revascularisation), and TVR (target vessel revascularization), stent thrombosis |
| Giles.2008 | proxy | CV mortality and hospitalization or ER (emergency room) visit for HF |
| PPAR.Study | NA |  |
| PROactive | proxy | Death, non-fatal MI excluding silent MI, stroke |
| PROFIT-J | proxy | All death, non-fatal stroke, non-fatal MI |
| RECORD | MACE | CV death, MI, or stroke |
| SAOR.  TIMI.53 | MACE | CV death, non-fatal MI, or non-fatal ischemic stroke |
| SPREAD-DIMCAD | proxy | Non-fatal MI, non-fatal stroke or arterial revascularization by percutaneous transluminal coronary angioplasty (PTCA) or by coronary artery bypass graft, death from a CV cause, and death from any cause |
| SUSTAIN.6 | MACE | Death from CV causes, non-fatal MI, or non-fatal stroke |
| TECOS | proxy | CV death, non-fatal MI, non-fatal stroke, or hospitalization for unstable angina |
| TIDE | MACE | MI, stroke or CV death |
| TOSCA.IT | proxy | All-cause mortality, non-fatal MI (including silent MI), non-fatal stroke, unplanned coronary revascularization |
| UGDP | NA |  |
| UKPDS.33 | NA |  |
| UKPDS.34a | NA |  |
