# Supplementary material for: GLUcose COntrol Safety & Efficacy in type 2 DIabetes, a systematic review and NETwork meta-analysis
Source: PLoS One. 2019 Jun 25;14(6):e0217701. doi: 10.1371/journal.pone.0217701 (PMC6592598; doi:10.1371/journal.pone.0217701)
Supplement: S3 Table — (DOCX) [file pone.0217701.s007.docx]

**S3 Table. Reported definitions of the serious adverse event (SAE) outcome used for each trial.** AE: adverse event. SAE: serious adverse events

| **Study** | **Severe/serious adverse events reported definitions** |
| --- | --- |
| ADVANCE | Hospitalization |
| APPROACH | NA |
| CANVAS | NA (Event rate per 1000 patient/year for the CANVAS Programme) |
| CANVASR | NA (Event rate per 1000 patient/year for the CANVAS Programme) |
| CARMELINA | NA |
| COSMIC | Any fatal, life-threatening, permanently or substantially disabling events, resulted in permanent or significant disability or incapacity, required or prolonged hospitalization, important event that jeopardized the patient or required intervention to prevent a serious outcome, a congenital abnormality, a cancer, an overdose of medication, or drug dependency or drug abuse. |
| DECLARE TIMI | Serious adverse event |
| ELIXA | MedDRA version 15.0 |
| EMPAREG | Any SAE |
| EXAMINE | Any SAE |
| EXSCEL | Any SAE |
| HARMONY | Serious Adverse Events by System Organ Class in subjects who took at least one dose of study drug in Appendix (Malignancies included in  Neoplasms) |
| HOME | NA |
| J-SPIRIT | NA |
| LEADER | SAE |
| ORIGIN | Aside from hypoglycemia and cancer, SAE were captured if considered related to a study drug. |
| PERISCOPE | Total, SAE |
| Kaku.2009 | "Other SAE" |
| Lee.2013 | NA |
| Giles.2008 | SAE (>1.5%) |
| PPAR.Study | NA |
| PROactive | Any SAE, non-endpoint events |
| PROFIT-J | NA |
| RECORD | NA |
| SAVOR.TIMI.53 | NA |
| SPREAD-DIMCAD | NA |
| SUSTAIN.6 | SAE (death, a life-threatening episode, hospitalization or prolongation of existing hospitalization, a persistent or substantial disability or incapacity, or an event otherwise considered to be an important medical event) |
| TECOS | NA |
| TIDE | Total, Serious Adverse Events |
| TOSCA.IT | SAE (death, a life-threatening episode, hospital admission or prolongation of existing hospital admission, or a persistent or substantial disability) |
| UGDP | NA |
| UKPDS.33 | NA |
| UKPDS.34a | NA |
| UKPDS.34b | NA |
