# Supplementary material for: GLUcose COntrol Safety & Efficacy in type 2 DIabetes, a systematic review and NETwork meta-analysis
Source: PLoS One. 2019 Jun 25;14(6):e0217701. doi: 10.1371/journal.pone.0217701 (PMC6592598; doi:10.1371/journal.pone.0217701)
Supplement: S4 Table — (DOCX) [file pone.0217701.s008.docx]

**S4 Table. Baseline cardiovascular (CV) risk groups (Grp) for the sensitivity analysis.** ‘H’ stands for “high cardiovascular risk at baseline”, ‘L’ for “low cardiovascular risk at baseline”. ACS: acute coronary syndrome, STEMI: ST elevation myocardial infarction, NSTEMI: non- ST elevation myocardial infarction, AP: angina pectoris, PCI: percutaneous coronary intervention, CABG: coronary artery bypass graft, TIA: transient ischemic attack, MI: myocardial infarction.

| Study | CV Grp | CV history (%) | CV history definitions |
| --- | --- | --- | --- |
| ELIXA | H | 99.8 | Qualifying ACS event: NSTEMI, STEMI, Unstable angina |
| EXAMINE | H | 99.7 | Myocardial infarction, Unstable angina requiring hospitalization |
| Pio.post.stent.Lee.2013 | H | 99.2 | Stable AP, Unstable AP, NSTEMI, STEMI |
| EMPAREG | H | 99 | Established cardiovascular disease |
| PROactive | H | 85.4 | Previous myocardial infarction, Previous stroke, Symptomatic peripheral arterial obstructive disease |
| SAVOR.TIMI.53 | H | 78.6 | Established atherosclerotic disease |
| TECOS | H | 74 | Prior cardiovascular disease (Myocardial infarction, >50% coronary stenosis, Prior PCI, CABG) |
| EXSCEL | H | 73.1 | Prior CV event at randomization |
| LEADER | H | 72.4 | Established cardiovascular disease |
| CANVAS1 | H | 72.2 | History of atherosclerotic vascular disease in CANVAS_P |
| CANVASR | H | 72.2 | History of atherosclerotic vascular disease in CANVAS_P |
| HARMONY | H | 70.6 | *Any of myocardial infarction, coronary artery bypass grafting, percutaneous coronary intervention, or at least 50% stenosis of coronary artery on angiography |
| PioGLy.mCD.GILES.2008 | H | 68.1 | NA |
| SUSTAIN.6 | H | 58.8 | established cardiovascular disease without chronic kidney disease |
| ORIGIN_tot | H | 58.8 | Prior cardiovascular event |
| SPREAD-DIMCAD | H | 58.6 | History of myocardial infarction |
| CARMELINA | H | 57 | Defined as albuminuria and prevalent macrovascular disease |
| DECLARE.TIMI.58 | L | 40.6 | 17,160 patients, including 10,186 without atherosclerotic cardiovascular disease |
| APPROACH | L | 38 | A total of 38% presented with acute coronary syndrome |
| TIDE | L | 34.5 | Cardiovascular disease |
| ADVANCE | L | 32.2 | History of major macrovascular disease |
| HOME | L | 31.7 | HOME 2002 : Diabetic complications Cardiovascular : 31.7% |
| PROFIT-J | L | 31.6 | silent cerebral infarction. Ohter previous CV disease: unknown |
| PERISCOPE | L | 28.2 | Prior myocardial infarction |
| RECORD | L | 17.4 | Ischaemic heart disease at baseline |
| TOSCA.IT | L | 11 | Previous cardiovascular disease |
| J-SPIRIT | L | 10 | Previous stroke (previous MI : 0) |
| UGDP | L | 10 | History of angina pectoris or of significant ECG abnormality |
| Pio.Kaku.2009 | L | 8.5 | History of CV events (Stroke excluding TIA, angina pectoris, myocardial infarction and coronary intervention procedure) |
| UKPDS.33 | L | NA | early diabetes |
| UKPDS.34a | L | NA | early diabetes |
| UKPDS.34b | L | NA | early diabetes |
| COSMIC | L | NA | type 2 diabetic patients suboptimally controlled on diet or sulfonylurea |
| PPAR.Study | NA | NA | NA |
| Mean |  | 54.9 |  |
| S.D |  | 29.4 |  |
| Median |  | 58.8 |  |
